# Genotypic and phenotypic characterisation of asymptomatic bacteriuria (ABU) isolates displaying bacterial interference against multi-drug resistant uropathogenic *E. Coli*

**DOI:** 10.1007/s00203-024-04114-0

**Published:** 2024-09-09

**Authors:** Ciara Kenneally, Craig P. Murphy, Roy D. Sleator, Eamonn P. Culligan

**Affiliations:** Department of Biological Sciences, Munster Technological University, Cork, T12 P928 Bishopstown Ireland

**Keywords:** Asymptomatic Bacteriuria, *E. Coli*, Urinary microbiome, Urobiome, Uropathogenic *E. Coli*, UTI

## Abstract

*Escherichia coli* can colonise the urogenital tract of individuals without causing symptoms of infection, in a condition referred to as asymptomatic bacteriuria (ABU). ABU isolates can protect the host against symptomatic urinary tract infections (UTIs) by bacterial interference against uropathogenic *E. coli* (UPEC). The aim of this study was to investigate the genotypic and phenotypic characteristics of five ABU isolates from midstream urine samples of adults. Comparative genomic and phenotypic analysis was conducted including an antibiotic resistance profile, pangenome analysis, and a putative virulence profile. Based on the genome analysis, the isolates consisted of one from phylogroup A, three from phylogroup B2, and one from phylogroup D. Two of the isolates, PUTS 58 and SK-106-1, were noted for their lack of antibiotic resistance and virulence genes compared to the prototypic ABU strain *E. coli* 83,972. This study provides insights into the genotypic and phenotypic profiles of uncharacterised ABU isolates, and how relevant fitness and virulence traits can impact their potential suitability for therapeutic bacterial interference.

**Supplementary Information:**

The online version contains supplementary material available at 10.1007/s00203-024-04114-0.

## Introduction

Currently, the standard definition of a urinary tract infection (UTI) is a bacterial infection that occurs in any part of the urinary system (Bono et al. 2022). This definition is ambiguous as it does not account for acute uncomplicated cystitis or asymptomatic bacteriuria (ABU) (Finucane 2017). ABU arises when two consecutive urine samples of the same bacterial isolate (≥ 10^5^ CFU/ml) are identified from individuals lacking symptoms of infection (Nicolle et al. 2005). Occurrence of ≥ 10^5^ CFU/ml bacteriuria also denotes a UTI in symptomatic individuals (Finucane 2017). Equivocal interpretations of UTIs means that the prescription of broad-spectrum antibiotics for these two conditions is common, yet often, not beneficial (Falagas et al. 2009; Gágyor et al. 2015). This relates to the previous doctrine that urine is sterile, and the presence of bacteria with or without symptoms indicates a state infection (Magistro and Stief 2019). Recent studies have disproved this theory, with the identification of a ‘core’ urinary microbiome (urobiome) that is currently the subject of considerable investigation (Ammitzbøll et al. 2021; Jones et al. 2021; Kenneally et al. 2022; Flores et al. 2023).

Uropathogenic *Escherichia coli* (UPEC) are a group of pathogenic strains that are attributable to conditions such as urinary incontinence, symptomatic UTIs (cystitis and pyelonephritis), and can lead to complications causing renal failure and urosepsis (Bien et al. 2012; Terlizzi et al. 2017). *E. coli* strains are also the main representatives associated with ABU (Dobrindt et al. 2016). Long term care facilities represent a hotspot for the presence of ABU strains, with the prevalence ranging from 25 to 50% in females and 15–40% in males, compared to incidences of 1–5% in younger cohorts (Nicolle 2016). Healthcare providers frequently struggle to differentiate ABU from urinary pathogens when individuals present with nonspecific symptoms, especially in the case of institutionalised patients (Cortes-Penfield et al. 2017). In turn, this has led to unnecessary prescription of antibiotics which has contributed to the evolution of multidrug resistant (MDR) *E. coli* (Cai et al. 2012, 2015). Currently, the European Association of Urology (EAU) guidelines prohibit treatment of ABU with the exception of two subgroups; pregnant woman and individuals undergoing surgical procedures involving breach of the bladder mucosa (Bonkat et al. 2020, 2022).

Only two ABU strains, *E. coli* 83,972 and *E. coli* HU2117 (Darouiche et al. 2010; Rudick et al. 2014; Packiriswamy et al. 2017; Schulz et al. 2020), are well characterised in the literature, with *E. coli* 83,972 being the prototypic ABU strain, colonising the bladder of an asymptomatic young girl for approximately three years (Lindberg et al. 1975; Andersson et al. 1991). An engineered derivative, *E. coli* HU2117, has been genetically modified with a deletion of *papG* as a safety measure to ensure P-fimbriae are not encoded to prevent adhesion to urothelium (Hull et al. 2002). *E. coli* 83,972 causes bacterial interference against UPEC and is the only ABU strain approved as a prophylactic treatment, for patients with lower urinary tract disfunction (LUTD) to prevent recurrent UTIs (rUTIs) (Bonkat et al. 2020). Bacterial interference is described as the use of low-virulence bacteria to compete against, inhibit colonisation, and prevent infection caused by disease-causing organisms (Darouiche and Hull 2012). In our previous study, *E. coli* 83,972 exhibited decreased efficacy compared to uncharacterised ABU isolates when testing antimicrobial activity against a bank of MDR UPEC (Kenneally et al. 2024).

Specifically, this work aimed to identify an ABU isolate of low virulence. Herein, we describe the genotypic and phenotypic characteristics of uncharacterised ABU isolates that previously displayed efficacious bacterial interference against a panel of MDR UPEC strains.

## Materials and methods

### *In vitro* characterisation

#### Bacterial strains and culture conditions

ABU isolates (PUTS 37, PUT 58, PUTS 59, S-07-4, and SK-106-1) were provided by Forsyth et al. (2018) and Leihof et al. (2021). Isolates from Forsyth et al. (2018) were obtained from midstream urine samples of healthy females between 18 and 40 years old. ABU isolates from Leihof et al. (2021) were obtained from midstream urine samples of individuals residing in nursing homes, ≥ 60 years of age. The ABU isolates were chosen for characterisation on the basis that they exhibited antimicrobial activity against UPEC in a previous study (Kenneally et al. 2024). *E. coli* ABU isolates were routinely cultured in Lysogeny Broth (LB; Neogen, Dublin, Ireland) at 37 °C for 18–24 h with continuous shaking or grown on LB agar (Neogen). All *E. coli* stocks were stored at − 20 °C and − 80 °C in 80% glycerol for further characterisation.

#### Confirmatory biochemical tests

Biochemical and morphological profiling were first conducted with the ABU isolate to confirm they were indeed *E. coli.* Colonies were grown on MacConkey (Thermo Fisher Scientific, Cork, Ireland) and Eosin Methylene Blue (EMB; Thermo Fisher Scientific) agar, and the appearance was analysed after Gram-staining (Thermo Fisher Scientific). Biochemical properties were assessed through oxidase (Merck, Dublin, Ireland) and catalase (Sigma-Aldrich, Wicklow, Ireland) testing.

#### Antibiotic susceptibility

Antibiotic susceptibility profiles of the ABU isolate were evaluated against clinically relevant antibiotics. Susceptibility profiling was conducted according to the Kirby-Bauer disk diffusion method at 37 °C for 24 h (CLSI, 2012). Overnight cultures were diluted to a 1.5 × 10^8^ CFU/mL suspension (0.5 McFarland standard) in sterile LB broth and spread over the surface of Mueller-Hinton agar (Merck, Dublin, Ireland) to achieve a full lawn of growth. Once dried, sterile forceps were used to place antibiotic discs on top of the agar plate. Following a 24 h incubation at 37 °C, zones of inhibition were measured with a vernier callipers and analysed as defined by EUCAST (2023), referring to the Enterobacterales breakpoints. *E. coli* ATCC 25,922 was used as a reference strain. Disc diffusions were completed in triplicate.

#### Motility assay

The motility assay was conducted as per the protocol described by Roos et al. (2006), with modifications. Briefly, LB agar plates were prepared containing 0.3% w/v agar. Overnight cultures were diluted to 1.5 × 10^8^ CFU/mL and 2 µL of the standardised *E. coli* cultures were stab pipetted in the centre of the agar plate before incubation at 37 °C for 24 h. Swimming motility was determined using a vernier callipers to measure the diameter from the centre of inoculation to determine the zone of migration.

#### Haemolytic activity

Columbia blood agar plates (Merck, Wicklow, Ireland) were used to determine the haemolytic activity of the *E. coli* isolates. Fresh overnights of ABU isolates were streaked onto separate Columbia blood agar plates. Haemolytic activity was visualised after 24 h incubation at 37 °C under anaerobic conditions. *Streptococcus agalactiae* MTU 4 was used as a positive control.

#### Biofilm formation

Biofilm formation was performed according to the microtiter plate method with crystal violet (O’Toole 2010; Whelan et al. 2020b). Briefly, overnight cultures of each isolate were prepared in LB and incubated at 37 °C for 16–18 h. Subsequently, cultures were standardised to a population of 1.5 × 10^8^ CFU/mL in LB. In a 96-well microtiter plate, 2 µL of each culture was added to three wells containing 198 µL of LB broth supplemented with 1% w/v glucose. *E. coli* ATCC 25,922 was used as a positive control. Uninoculated LB broth supplemented with 1% w/v glucose was used as a sterility control and 200 µL was placed into three wells. Plates were incubated at 37 °C for 24 h. The plate was turned upside down and gentle tapping was performed to discard unbound bacteria. Wells were washed three times with 200 µL of sterile phosphate buffered saline (PBS; Sigma Aldrich). Afterwards, attached cells were heat fixed at 50 °C for 1 h. Subsequently, 200 µL of crystal violet (1% v/v) was added into each well and the plates were incubated for 20 min at room temperature. Excess stain was removed, and wells were washed with PBS. Plates were left to dry for 30 min. Biofilm-bound crystal violet was solubilised with 200 µl of 30% v/v glacial acetic acid and incubated at room temperature for 20 min. Biofilms were quantified at OD_590_ in a microtiter plate reader. ABU isolates were categorised based on their biofilm formation as described by Whelan et al. (2020) whereby the average biofilm formation for the control strain *E. coli* ATCC 25,922 was allocated a score of 1. ABU isolates were given a proportional score whereby a score of 0.5 equals to 50% of the control. Isolates scoring between 0.9 and 2.0 are strong biofilm formers. Scores between 0.4 and 0.9 are classified as weak biofilm formers, and less than 0.4 indicates indeterminate biofilm formers.

### *In silico* genome analysis

#### Pangenome analysis

In addition to the ABU genomes sequenced through Enhanced Sequencing (both Illumina and Oxford Nanopore sequencing) by Microbes NG (Birmingham, United Kingdom) in our previous study (Kenneally et al., 2022), a further 68 complete and draft ABU and UTI *E. coli* genomes (Supplementary Table 1) were downloaded from GenBank as reference strains, including 66 from a study by Garretto et al. (2020). Genomes were annotated with PROKKA (v. 1.14.6) (Seemann 2014) under default parameters. Core and accessory genes within each genome were determined using Roary (v. 3.13) (Page et al. 2015). Coding sequences with 95% identity were considered as part of the core/accessory genome (Horesh et al. 2021), with a core genome being defined as genes present within at least 99% of samples (Page et al. 2015; Lynch et al. 2021). FASTTREE 2.10.1 (Price et al. 2010) was used to construct a maximum-likelihood phylogenetic tree based on the core genome alignment from Roary and iTOL (v6) (Letunic and Bork 2021) was used to visualise the resulting trees. The generated gene absence and presence table was analysed for gene co-occurrences (associations) and avoidance (dissociations) using Coinfinder (v 1.2.1) (Whelan et al. 2020b), applying the default significance threshold of 0.05 and employing a Bonferroni correction.

#### Genomic characterisation

Serotypes were assigned to the ABU isolates after genomes were analysed on SerotypeFinder 2.0 (Joensen et al. 2015). ABU isolates were categorised into phylogroups using EzClermont 0.7 (Waters et al. 2020). Multilocus sequence types (MLST) were determined on cgMLSTFinder 1.2 (Zhou et al. 2020). The identification of plasmid replicon sequences was performed using PlasmidFinder 2.1 with default parameters (threshold ID = 95%; maximum coverage = 60%) (Carattoli et al. 2014). Mobile elements and insertion sequences were identified using MobileElementFinder (Johansson et al. 2021). PHASTER was used to detect prophage elements (Arndt et al. 2016). Genomes were screened for *fimH* and *fumC* alleles on CHtyper 1.0 (Roer et al. 2018). Antimicrobial resistance genes were detected both in the genome files and the extracted plasmid sequences using ABRicate (v 0.9.8) (https://github.com/tseemann/abricate) employing the ResFinder (Florensa et al. 2022) and Comprehensive Antibiotic Resistance Database (CARD) (Alcock et al. 2023) databases.

Genomes were searched for virulence factors as previously described by Morales et al. (2023). The list of virulence factors, curated by Morales et al. (2023), included diarrheagenic virulence factors from the VirulenceFinder database and uropathogenic-associated virulence factors described in the literature. BLASTN (https://blast.ncbi.nlm.nih.gov/Blast.cgi) was used to search for virulence factor sequences in each genome applying a threshold of 70% identity and 85% alignment to consider a virulence factor as present, in accordance with (Morales et al. 2023). Gene presence was visualised in Artemis (Carver et al. 2012) and manual annotation was conducted, where applicable, if genes had not previously been annotated.

## Results

### Phenotypic and biochemical characteristics

ABU isolates had typical *E. coli* biochemical characteristics (Table 1); Gram-negative, oxidase negative, and catalase positive isolates with adequate growth on EMB and MacConkey agar with lactose fermentation.

**Fig. 1:**
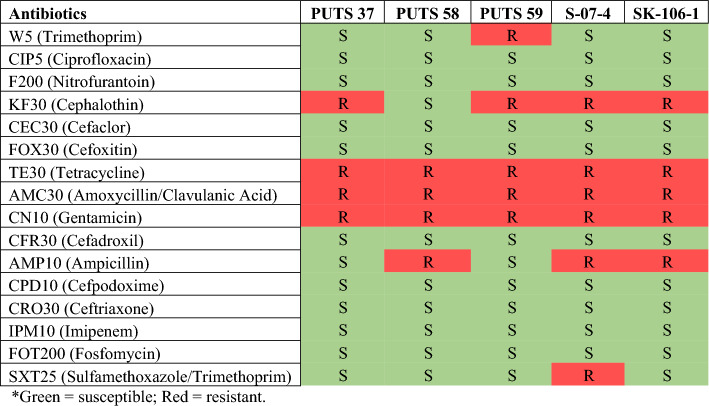
In vitro antibiotic susceptibility analysis of the ABU isolates PUTS 37, PUTS 58, PUTS 59, S-07-4, and SK-106-1

Antibiotic resistance profiles of the ABU isolates against 20 antibiotics which have been previously, or are currently, recommended as therapies for urogenital infections (Kot 2019; Bonkat et al. 2020, 2022) are presented in Fig. 1. All isolates were resistant to tetracycline, gentamicin, and amoxycillin/clavulanic acid. Isolates were sensitive to ciprofloxacin, nitrofurantoin, cefaclor, cefoxitin, cefadroxil, cefpodoxime, ceftriaxone, imipenem, and fosfomycin. S-07-4 was the most resistant ABU isolate, exhibiting resistance to six (37.5%) of the antibiotics, followed by PUTS 59 and SK-106-1 with resistance against five (31.3%) of the antibiotics. PUTS 58 and PUTS 37 were the least resistant isolates, displaying resistance to 4 (25%) antibiotics. S-07-4 appears to be a potential MDR isolate, however, further bioinformatic analysis was required to confirm this.

Motility tests on the ABU isolates revealed that *E. coli* PUTS 37 was non-motile, whilst *E. coli* PUTS 58 and PUTS 59 both showed low motility. Both *E. coli* S-07-4 and *E. coli* SK-106-1 were highly motile isolates (++), swarming the agar plates. None of the ABU isolates displayed β-haemolytic activity on the blood agar plates, appearing as either α- or γ-haemolytic (Table 1). All the ABU isolates exhibited weak biofilm formation capabilities (Table 1).

**Table 1:** Phenotypic characterisation of the ABU isolates

Isolate	Motility	Biofilm Formation	Haemolysis
PUTS 37	−	Weak	α
PUTS 58	+	Weak	γ
PUTS 59	+	Weak	α
S-07-4	++	Weak	α
SK-106-1	++	Weak	γ

### Pangenome analysis

**Fig. 2:**
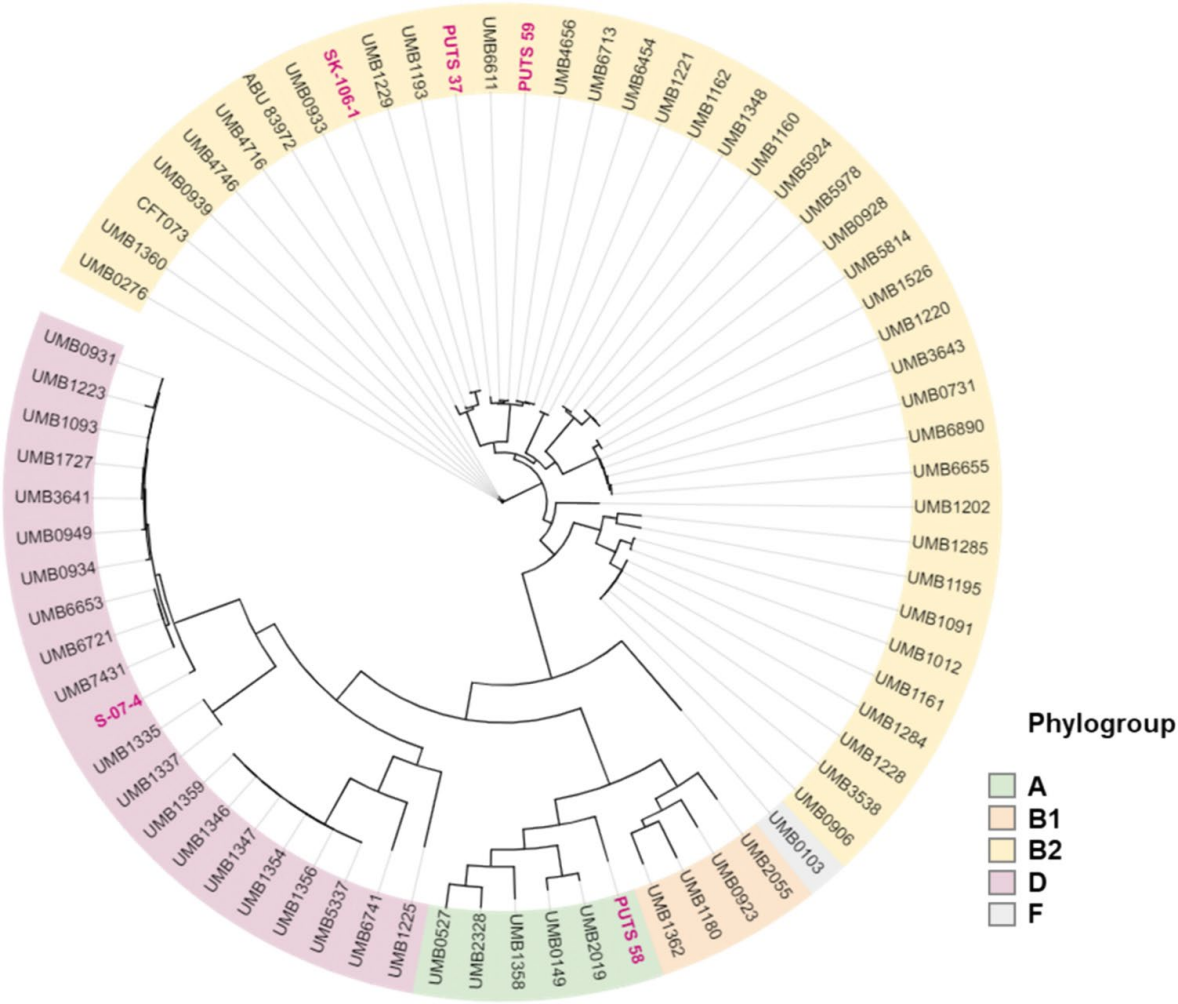
Maximum-likelihood phylogenetic tree of 73 *E. coli* isolates from the urinary tract. The maximum likelihood tree was generated on FASTTREE using the core genome alignment and was visualised using iTOL. The five ABU isolates from this study are indicated with pink font. Phylogroups are indicated by the coloured boxes, as identified through EzClermont

Pangenome analysis was performed on 73 genomes in total: consisting of the five uncharacterised ABU isolates (PUTS 37, PUTS 58, PUTS 59, S-07-4, and SK-106-1), the ABU and UPEC reference genomes of *E. coli* 83,972 and *E. coli* CFT073, respectively. An additional 66 *E. coli* genome sequences were included in the pangenome analysis (Fig. 2), representing *E. coli* strains from the urinary tract of individuals with and without symptoms of infection (Garretto et al. 2020). The entire pangenome consisted of 18,317 genes present within one or more of the *E. coli* genomes. The core and accessory genomes consisted of 3149 and 15,168 genes, respectively. Overall, approximately 30% of the pangenome consists of unique genes (5455 genes) which correlates with previous research showing *E. coli* having an open pangenome (Rasko et al. 2008; Tantoso et al. 2022). This is also further reinforced by the identification of over 1,000 new unidentified genes, suggesting that new genes are likely to be continually detected as new *E. coli* sequences are analysed. No correlation was detected between the presence/absence of genes in the pangenome.

### Genome characterisation

Genome sequence analysis indicated that three of the ABU isolates (*E. coli* PUTS 37, PUTS 59, and SK-106-1) were representatives of the B2 phylogroup (Table 2). *E. coli* PUTS 58 belongs to phylogroup A and *E. coli* S-07-4 is a member of phylogroup D. MLSTs varied amongst the five ABU isolates in accordance with the cgMLST *Escherichia coli* database, Enterobase (Zhou et al. 2020). *E. coli* PUTS 37 and *E. coli* PUTS 59 represented the same MLST, ST95, clustered together with avian pathogenic *E. coli* (APEC) and UPEC isolates. *E. coli* PUTS 58 was grouped into ST2795, a MSLT group containing few *E. coli* isolates thus far. *E. coli* PUTS 58 is clustered together with one known enterotoxigenic *E. coli* (ETEC) isolate, *E. coli* 1743. Additional *E. coli* isolates grouped into ST2795 in the literature are from marine environments (Kvesić et al. 2022). *E. coli* SK-106-1 belonged to ST12 and *E. coli* S-07-4 was grouped as ST69. ST12 has previously been associated with UPEC (Nüesch-Inderbinen et al. 2017; Drage et al. 2019), meanwhile, ST69 is an MLST group associated with the ‘pandemic UPEC clones’ (Yamaji et al. 2018).

All ABU isolates were predicted to harbour at least one plasmid (Table 3). The genome sequence of *E. coli* PUTS 58 was found to contain the most plasmids as ten plasmid replicon sequences were detected. Similar to previous studies, colicin-encoding plasmids (Col) and incompatibility plasmids (Inc) were most abundant in the ABU isolates (Garretto et al. 2020; Morales et al. 2023). *E. coli* S-07-4 contained an IncQ1 plasmid which was predicted to contain the antimicrobial resistance gene *bla*TEM-1B. *E. coli* PUTS 37 was the only ABU genome with a plasmid encoding a virulence factor, an observation typically associated with the B2 phylogroup. In this instance, the iron transport locus *sitA* was detected on a IncFIB plasmid.

Additional mobile genetic elements were identified in all five ABU genomes with MobileElementFinder. ABUs contained ≥ 14 mobile genetic elements (MGEs). MGEs present in *E. coli* PUTS 59 contained the most virulence genes, with a total of 14 virulence factors encoded by various MGEs. Whilst this observation is consistent with the B2 phylogroup, the ABU isolate representing the phylogroup A, *E. coli* PUTS 58, contained the most MGEs (*n* = 28) encoding the fewest number of virulence-associated genes (*n* = 0). Prophage elements were found in all isolates, with enterobacterial phages the most abundant prophages identified; however, *E. coli* S-07-4 contained no complete sequences. *E. coli* PUTS 37 contained the most complete prophage elements, whilst *E. coli* SK-106-1 contained the least (Table 4). Thus, no correlation to the B2 phylogroup was observed.

Antimicrobial resistance gene predictions showed that every ABU isolates contains the *mdfA* (multidrug efflux) gene (Table 5). The phenotypic analysis did not correlate with the genotypic profile for isolate PUTS 37, PUTS 58, PUTS 59, and SK-106-1 as they were lacking in additional resistance genes. *E. coli* S-07-4 was the only ABU isolate analysed that contained additional resistance genes. In total, 12 resistance genes were present in the S-07-4 genome, further supporting that S-07-4 can be defined as a MDR isolate. .

**Table 2:** Genome-based characterisation of the ABU isolates

Isolate	Phylogroup	Serotype	Sequence type	Intact prophage	Plasmid Replicons	MGEs
PUTS 37	B2	O2:H4	ST 95	8	2	13
PUTS 58	A	O160:H27	ST 2795	4	10	28
PUTS 59	B2	O18:H7	ST 95	6	3	14
S-07-4	D	nt: H18	ST 69	0	1	14
SK-106-1	B2	O4:H5	ST 12	1	1	14

**Table 3:** Plasmid replicons identified in the ABU genomes

Isolate	Plasmid Replicons
PUTS 37	IncFIB, IncFIC
PUTS 58	Col440I, pENTAS02, Col440I, ColpVC, ColRNAI, IncHI1B, IncHI1A, IncN, IncFIA, Col156
PUTS 59	IncFIB, Col156, IncFII
S-07-4	IncQ1
SK-106-1	IncBOKZ

**Table 4:** Intact prophages identified in the ABU genomes

Isolate	Intact Prophages
PUTS 37	*Escherichia* phage STX2, *Escherichia* phage pro483, Enterobacteria phage λ, Enterobacteria phage cdtI, Enterobacteria phage λ, Enterobacteria phage cdtI, Enterobacteria phage Sf101, *Shigella* phage SfII
PUTS 58	Enterobacteria phage phiT5282H, Enterobacteria phage λ, *Klebsiella* phage ST512, *Escherichia* phage RCS47
PUTS 59	*Shigella* phage SfII, Enterobacteria phage Sf101, Enterobacteria phage λ, Enterobacteria phage λ, *Burkholderia* phage phiE255, *Escherichia* phage STX2
SK-106-1	Enterobacteria phage λ

**Table 5:** Antimicrobial resistance (AMR) genes identified in the ABU isolates

AMR genes	PUTS 37	PUTS 58	PUTS 59	S-07-4	SK-106-1
aph(6)-Id	−	−	−	+	−
aph(3″)-Ib	−	−	−	+	−
ant(3″)-Ia	−	−	−	+	−
aadA1	−	−	−	+	−
aadA5	−	−	−	+	−
blaTEM-1B	−	−	−	+	−
dfrA1	−	−	−	+	−
mdf (A)	+	+	+	+	+
mph(B)	−	−	−	+	−
sul1	−	−	−	+	−
sul2	−	−	−	+	−
tet(A)	−	−	−	+	−

**Fig. 3:**
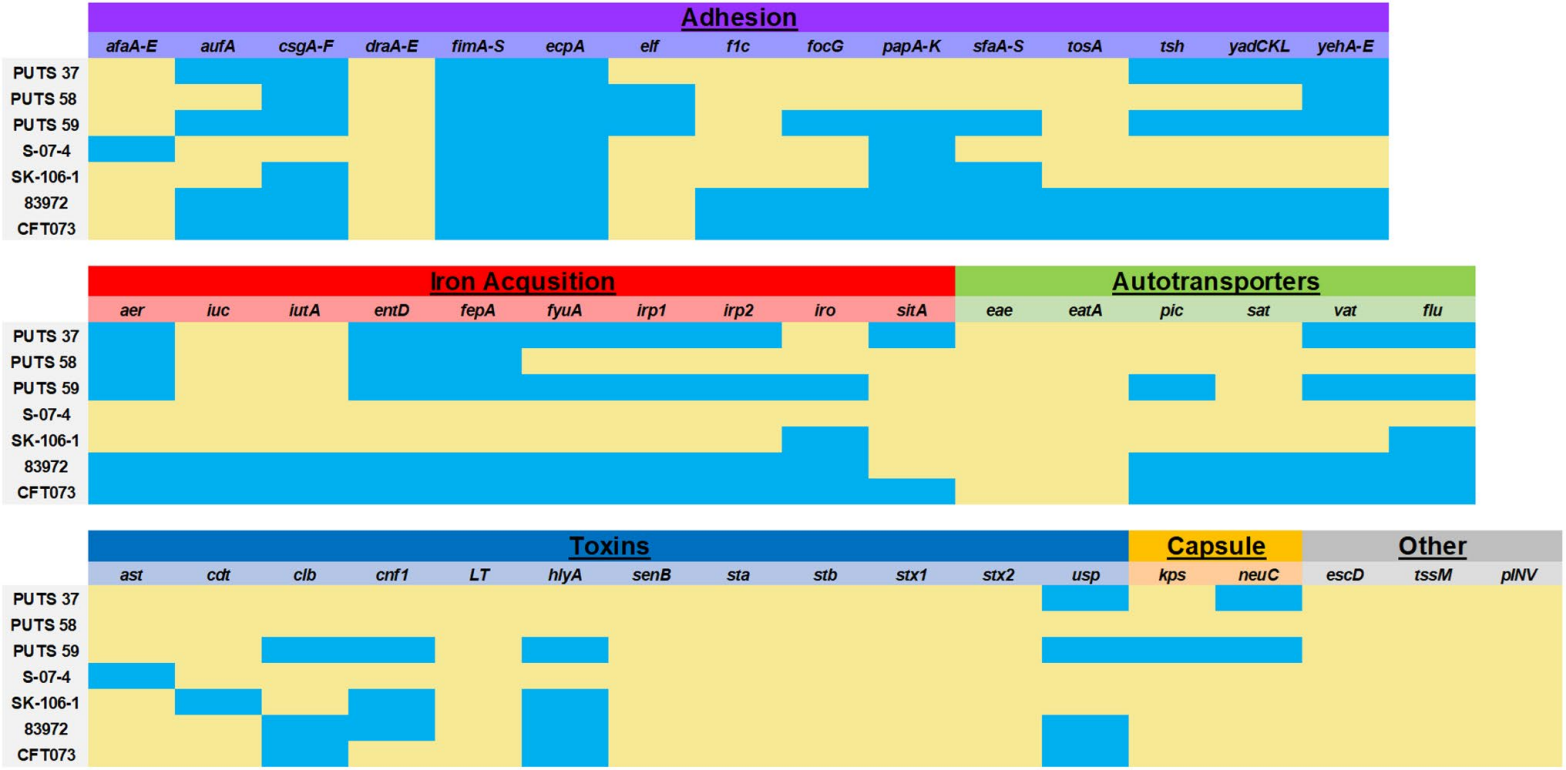
Heatmap indicating the presence or absence of virulence genes. The heatmap is based on the nucleotide percentage identity of virulence genes determined by BLASTN. Virulence genes are grouped according to function. Blue indicates the presence of a gene, yellow indicates the absence of a gene. Reference genomes included the ABU strain *E. coli* 83,972 and UPEC strain *E. coli* CFT073

The presence of virulence factors in the ABU isolates was compared to two reference genomes, the ABU strain *E. coli* 83,972 and the UPEC strain *E. coli* CFT073 (Fig. 3). *E. coli* PUTS 59 was most similar to *E. coli* 83,972, lacking only five of the virulence factors that the prototypic strain encodes. The two other members of the B2 phylogroup, *E. coli* PUTS 37 and *E. coli* SK-106-1 vastly differed in the virulence factors they encode when analysed against PUTS 59. Of particular note were virulence factors associated with iron acquisition and adhesion. Virulence factors present in PUTS 37 are similar to PUTS 59, however, eight virulence genes that were identified in PUTS 59 were not present in PUTS 37. Additionally, SK-106-1 contains nine virulence factors that were also present in PUTS 59. *E. coli* S-07-4 contained the least amount of virulence factors, with only five being identified in the entire genome (Table 6).

*E. coli* S-07-4 contained no iron acquisition genes (Table 6). Iron acquisition genes present in *E. coli* PUTS 58 encode the enterobactin siderophore system. PUTS 37 and PUTS 59 also contained the enterobactin siderophore genes, along with genes required for yersiniabactin production. PUTS 59 was the only ABU isolate to also encode the salmochelin siderophore uptake system. When salmochelin is encoded with colibactin and a bacteriocin, this typically can point to an *E. coli* isolate having the potential to become a more invasive pathogen (Massip et al. 2020). Colibactin (*clb*) was present only in *E. coli* PUTS 59. Chaperone-usher fimbriae determinants were found in every ABU isolate. Type 1 fimbriae and *E. coli* common pilus (ECP) genes were encoded by all five ABU isolates. P-fimbriae determinants were encoded by *E. coli* PUTS 59, S-07-4, and SK-106-1, whilst F1C fimbriae were not present in any of the new ABU isolates analysed in this study.

**Table 6:** Abundance of virulence factors in ABU isolates and their frequency on plasmids and mobile genetic elements

Isolate	Virulence factors	Adhesion	Iron acquisition	Toxins	Plasmid encoded	MGE encoded
PUTS 37	18	7	7	1	1	6
PUTS 58	8	4	3	0	0	0
PUTS 59	27	9	7	4	0	14
S-07-4	5	4	0	1	0	1
SK-106-1	10	5	1	1	0	6

## Discussion

*E. coli* is a versatile bacterium that colonises the urogenital tract, and remains the most common organism isolated from females (Klein and Hultgren 2020; Price et al. 2020). The severity of *E. coli*-mediated infections depends on the virulence potential of the specific strain, together with the robustness of the host’s immune response. ABU isolates are typically associated with encoding fewer virulence factors than cystitis- or pyelonephritis-causing UPEC, and have been proposed to have a fitness advantage over pathogenic strains (Watts et al. 2010; Stork et al. 2018). Bacteria deemed suitable for use as probiotics are required to be of low-virulence and contain no antibiotic resistance genes. Our previous study highlight the antimicrobial activity of five ABU strains against UPEC (Kenneally et al. 2022).This study, therefore, aimed to phenotypically and genotypically characterise the five ABU isolates, and determine their suitability as live biotherapeutics to establish bacterial interference against UPEC. *E. coli* 83,972 has received much attention due to its success as a prophylactic for individuals suffering from LUTD, establishing bacterial interference (Bonkat et al. 2022). This study presents five ABU isolates with low in vitro virulence properties and which, in general, contain fewer virulence factors than *E. coli* 83,972.

ABU isolates typically fall into two categories; isolates that resemble commensal *E. coli* belong to the phylogroups A and B1 have fewer virulence genes, and ABU *E. coli* which resemble UPEC belong to phylogroup B2 and D (Biggel et al. 2020). Putative virulence factors are mostly found in phylogroup B2 strains, however these are not always linked to pathogenicity. Highly virulent, extensively characterised UPEC strains, CFT073 and UTI89, are B2 members (Hogins et al. 2023). *E. coli* 83,972 also belongs to the B2 group, however, many of the virulence factors in 83,972 are inactive due to point mutations (Klemm et al. 2007; Zdziarski et al. 2008, 2010). Four of the ABU isolates in this study appear more closely related to UPEC; PUTS 37, PUTS 59, and SK-106-1 belong to phylogroup B2 and S-07-4 to phylogroup D. The allocation of MLSTs to these isolates correlated with the phylogroups. In particular, ST95 (PUTS 37 and PUTS 59) and ST69 (S-07-4) have both been referred to as pandemic MLSTs due to high levels of multidrug resistant UPEC strains identified among these ST’s (Yamaji et al. 2018; Tarlton et al. 2019; de Souza da-Silva et al. 2020). Meanwhile, PUTS 58 appears to resemble a commensal, categorised as phylogroup A and contains fewer putative virulence factors than *E. coli* 83,972.

The putative avirulent nature of the investigated ABU isolates may result from the presence of dormant virulence factors. Overall, PUTS 37, PUTS 58, S-07-4, and SK-106-1 contained few adhesins compared to *E. coli* 83,972, while PUTS 59 more closely resembled the virulence profile. Here, *fim* and *ecpA* were the only two putative virulence factors identified in every isolate. In particular, *fim* genes are required to express type 1-fimbriae. Deletion and mutations of *fim* genes in UPEC does hinder the ability to colonise the bladder (Spaulding et al. 2017), however, the *fim* genes are highly conserved in commensal strains enhancing colonisation (Watts et al. 2010; Chevalier et al. 2021). The *ecpA* gene is also widely distributed among commensal and pathogenic strains, enhancing cell adherence and, contrary to our results, biofilm formation (Saldaña et al. 2009, 2014). Interestingly, a recent study by Naziri et al. (2021) determined that the difference between weak and strong biofilm formers often resulted in the presence of *sfa* genes in UPEC. PUTS 59 and SK-106-1 were the only two ABU isolates containing the *sfa* gene, which possibly contributed to the weak biofilm formation observed. Thus, the investigated ABU isolates appear to be of low-virulence compared to clinically relevant UPEC (Davari Abad et al. 2019; Naziri et al. 2021).

UPEC are known for having a complex virulence profile, resulting in latent reservoirs and subsequently incidence of rUTIs (Vagios et al. 2020). In contrast, ABU isolates can successfully reduce UPEC levels and establish long-term persistence due to the competition for attachment sites and nutrients (Darouiche and Hull 2012). Yersiniabactin, salmochelin, and aerobactin siderophores play a role in iron uptake, important for ABU growth in urine (Frick-Cheng et al. 2020; Massip et al. 2020). PUTS 59 contains both the salmochelin, yersiniabactin and aerobactin siderophore genes, along with enterobactin producing genes. PUTS 37 lacks the salmochelin genes and PUTS 58 contains only the aerobactin and enterobactin genes. Production of different siderophores can make a bacterium more adaptive, as siderophores are maximally produced at different pH levels and in different carbon sources (Valdebenito et al. 2006). The production of a variety of siderophores in urogenital *E. coli* is likely a competitive trait as urine can span a wide pH range of ~ 4.5–8.0, and contains limited carbon and iron (Reitzer and Zimmern 2020). Furthermore, siderophores can contribute increased pathogenesis causing a bacterium to invade urethral cells, disrupt the iron homeostasis of competing bacteria, and induce mitochondrial autophagy (Khasheii et al. 2021).

Of potential concern, PUTS 59 encodes colibactin, a virulence factor that appears to have a functional synergy with salmochelin; promoting colonisation, if not also enhancing virulence (Massip et al. 2020). Furthermore, PUTS 59 produces the pore-forming toxin, HlyA, which can alter epithelial cells, disable macrophages, and supress inflammatory responses, ultimately causing serious tissue damage (Dhakal and Mulvey 2012; Wang et al. 2020). PUTS 59 has the most similar virulence profile to *E. coli* CFT073 and 83,972. S-07-4, on the other hand, contains the fewest virulence genes, but has the most antibiotic resistance genes.

Antibiotic treatment of ABU strains is rarely beneficial, and is associated with higher rates of rUTIs and antimicrobial resistance (Cai et al. 2012, 2015). The *mdfA* gene was present in every ABU isolate analysed in this study. This gene encodes the MdfA multidrug efflux protein (Yardeni et al. 2020), conferring resistance to a broad spectrum of antibiotics including chloramphenicol, tetracycline, erythromycin, and fluoroquinolones (Edgar and Bibi 1997; Suarez and Martiny 2021). *E. coli* S-07-4 was the only isolate that harboured more than one resistance gene, containing a total of twelve. S-07-4 has the potential to be resistant to multiple drugs, including aminoglycosides, fluroquinolones, sulphonamides, tetracyclines, cephalosporins, and carbapenems (Ashenafi et al. 2014; Hansen et al. 2019; Adekanmbi et al. 2020). The in vitro antimicrobial resistance screen revealed that the ABU isolates were susceptible to first-generation cephalosporins, a first-line treatment for UTIs. However, this study only included five ABU isolates and a previous meta-analysis representing 8 countries (Poland, Iran, Jordan, Mexico, China, India, Nigeria, and Brazil) has reported resistance rates against first-generation and third-generation cephalosporins to be 38.8% and 37.0%, respectively (Bunduki et al. 2021).

Interestingly, *E. coli* PUTS 58 and SK-106-1 were characterised as isolates with low in vitro virulence. *In silico* analysis supported this, as the virulence gene profile was less than that of *E. coli* 83,972 and CFT073. Furthermore, both isolates contained only one antibiotic resistance gene, further suggesting PUTS 58 and SK-106-1 could be suitable biotherapeutics. PUTS 58 and SK-106-1 have previously exhibited superior interference and antimicrobial activity against UPEC strains, compared to other investigated ABU isolates and *E. coli* 83,972 (Kenneally et al., 2022). In particular, PUTS 58 appears to resemble commensal *E. coli* and the presence of both aerobactin and enterobactin siderophores may contribute to the persistence of PUTS 58 in iron-limiting environments, such as urine. Our previous studies have also indicated that PUTS 58 cell free supernatant is stable in clinically relevant conditions such pH of 2.5–10 and temperatures of 4–70 °C (Kenneally et al., 2024).

The under-utilisation of ABU isolates, to date, can be ascribed to a lack of research on their safety profile and influence on the urobiome. This study was limited to the characterisation of five ABU isolates, as our research has identified that the five ABU isolatess display antimicrobial activity against UPEC (Kenneally et al. 2024). Furthermore, the full safety profile is still warranted as a limitation of this study was that the up/down-regulation of virulence genes in real-time was not able to be conducted. This will furtheranalyse the full safety profile and unveil a more comprehensive molecular profile of the ABU isolates, in particular, PUTS 58. Additionally, in vitro cell culture and in vivo analysis in bladder models to determine if PUTS 58 is durable when attaching to catheters and successfully inhibits uropathogens is warranted, as *E. coli* 83,972 satisfies this criteria for LUTD patients enduring rUTIs. Ultimately, further investigations could result in ABU isolates gaining a Generally Recognised as Safe (GRAS) status and be implemented as urobiome-based therapeutics.

## Supplementary Information

Below is the link to the electronic supplementary material.

Supplementary material 1 (DOCX 25.2 kb)

## Data Availability

Raw reads and assembled contigs are available under the accession numbers JBAKMN000000000, JBAKMO000000000, JBAKMP000000000, DAEBDQ000000000, DAEBEH000000000.
