# Supplementary material for: Genotypic and phenotypic characterisation of asymptomatic bacteriuria (ABU) isolates displaying bacterial interference against multi-drug resistant uropathogenic E. Coli
Source: Arch Microbiol. 2024 Sep 9;206(10):394. doi: 10.1007/s00203-024-04114-0 (PMC11381485; doi:10.1007/s00203-024-04114-0)
Supplement: Supplementary file 1 — Supplementary material 1 (DOCX 25.2 kb) [file 203_2024_4114_MOESM1_ESM.docx]

**Supplementary Table 1: E. coli strains used in the pangenome analysis of the ABU isolates.**

| Strain | Phylogroup | Infection | Accession no. (Genbank or SRA) |
| --- | --- | --- | --- |
| *Escherichia coli* PUTS 58 | A | ABU | JBAKMO000000000 |
| *Escherichia coli* PUTS 37 | B2 | ABU | JBAKMN000000000 |
| *Escherichia coli* PUTS 59 | B2 | ABU | JBAKMP000000000 |
| *Escherichia coli* SK-106-1 | B2 | ABU | DAEBDQ000000000 |
| *Escherichia coli* S-07-4 | D | ABU | DAEBEH000000000 |
| *Escherichia coli* strain UMB2019 | A | UUI | RRVF00000000 |
| *Escherichia coli* strain UMB1358 | A | UTI | RRVL00000000 |
| *Escherichia coli* strain UMB2328 | A | UUI | RRVC00000000 |
| *Escherichia coli* strain UMB0527 | A | OAB | RRWQ00000000 |
| *Escherichia coli* strain UMB0149 | A | OAB | RRWS00000000 |
| *Escherichia coli* strain UMB2055 | B1 | UUI | RRVE00000000 |
| *Escherichia coli* strain UMB1362 | B1 | UTI | RRVI00000000 |
| *Escherichia coli* strain UMB1180 | B1 | UTI | RRWD00000000 |
| *Escherichia coli* strain UMB0923 | B1 | UTI | RRWN00000000 |
| *Escherichia coli* strain UMB6713 | B2 | ABU | RRUK00000000 |
| *Escherichia coli* strain UMB6454 | B2 | ABU | RRUU00000000 |
| *Escherichia coli* strain UMB0939 | B2 | ABU | RRUR00000000 |
| *Escherichia coli* strain UMB6611 | B2 | ABU | RRUT00000000 |
| *Escherichia coli* strain UMB4716 | B2 | ABU | RRUT00000000 |
| *Escherichia coli* strain UMB4746 | B2 | ABU | RRUZ00000000 |
| *Escherichia coli* strain UMB0933 | B2 | ABU | RRWK00000000 |
| *Escherichia coli* strain UMB0928 | B2 | ABU | RRWM00000000 |
| *Escherichia coli* strain UMB6890 | B2 | UUI | RRUI00000000 |
| *Escherichia coli* strain UMB1221 | B2 | UTI | RRUG00000000 |
| *Escherichia coli* strain UMB3643 | B2 | UUI | RRUF00000000 |
| *Escherichia coli* strain UMB1161 | B2 | UTI | RRUP00000000 |
| *Escherichia coli* strain UMB6655 | B2 | UUI | RRUL00000000 |
| *Escherichia coli* strain UMB5978 | B2 | UTI | RRUV00000000 |
| *Escherichia coli* strain UMB5924 | B2 | UTI | RRUW00000000 |
| *Escherichia coli* strain UMB5814 | B2 | UUI | RRUX00000000 |
| *Escherichia coli* strain UMB1526 | B2 | UTI | RRVH00000000 |
| *Escherichia coli* strain UMB1348 | B2 | UTI | RRVO00000000 |
| *Escherichia coli* strain UMB1229 | B2 | UTI | RRVV00000000 |
| *Escherichia coli* strain UMB1220 | B2 | UTI | RRVZ00000000 |
| *Escherichia coli* strain UMB1202 | B2 | UTI | RRWA00000000 |
| *Escherichia coli* strain UMB1195 | B2 | UTI | RRWB00000000 |
| *Escherichia coli* strain UMB1091 | B2 | UTI | RRWG00000000 |
| *Escherichia coli* strain UMB1012 | B2 | UTI | RRWH00000000 |
| *Escherichia coli* strain UMB4656 | B2 | UTI | RRVA00000000 |
| *Escherichia coli* strain UMB3538 | B2 | UUI | RRVB00000000 |
| *Escherichia coli* strain UMB1360 | B2 | UTI | RRVJ00000000 |
| *Escherichia coli* strain UMB1285 | B2 | UTI | RRVT00000000 |
| *Escherichia coli* strain UMB1228 | B2 | UTI | RRVW00000000 |
| *Escherichia coli* strain UMB0906 | B2 | UTI | RRWO00000000 |
| *Escherichia coli* strain UMB1284 | B2 | UTI | RRVU00000000 |
| *Escherichia coli* strain UMB1162 | B2 | UTI | RRWE00000000 |
| *Escherichia coli* strain UMB0731 | B2 | OAB | RRWP00000000 |
| *Escherichia coli* strain UMB0276 | B2 | OAB | RRWR00000000 |
| *Escherichia coli* strain UMB1193 | B2 | UTI | RRWC00000000 |
| *Escherichia coli* strain UMB1160 | B2 | UTI | RRWF00000000 |
| *Escherichia coli* CFT073 | B2 | UTI | AE014075 |
| *Escherichia coli* ABU 83972 | B2 | ABU | CP001671 |
| *Escherichia coli* strain UMB6721 | D | UTI | RRUJ00000000 |
| *Escherichia coli* strain UMB6471 | D | UTI | RRUM00000000 |
| *Escherichia coli* strain UMB1093 | D | UTI | RRUQ00000000 |
| *Escherichia coli* strain UMB7431 | D | UTI | RRUH00000000 |
| *Escherichia coli* strain UMB3641 | D | UUI | RRUO00000000 |
| *Escherichia coli* strain UMB6653 | D | UTI | RRUS00000000 |
| *Escherichia coli* strain UMB5337 | D | UUI | RRUY00000000 |
| *Escherichia coli* strain UMB1727 | D | UUI | RRVG00000000 |
| *Escherichia coli* strain UMB1359 | D | UTI | RRVK00000000 |
| *Escherichia coli* strain UMB1354 | D | UTI | RRVN00000000 |
| *Escherichia coli* strain UMB1356 | D | UTI | RRVM00000000 |
| *Escherichia coli* strain UMB1347 | D | UTI | RRVP00000000 |
| *Escherichia coli* strain UMB1346 | D | UTI | RRVQ00000000 |
| *Escherichia coli* strain UMB1337 | D | UTI | RRVR00000000 |
| *Escherichia coli* strain UMB1223 | D | UTI | RRVY00000000 |
| *Escherichia coli* strain UMB0931 | D | UTI | RRWL00000000 |
| *Escherichia coli* strain UMB1335 | D | UTI | RRVS00000000 |
| *Escherichia coli* strain UMB1225 | D | UTI | RRVX00000000 |
| *Escherichia coli* strain UMB0949 | D | UTI | RRWI00000000 |
| *Escherichia coli* strain UMB0934 | D | UTI | RRWJ00000000 |
| *Escherichia coli* strain UMB0103 | F | OAB | RRWT00000000 |

*ABU: Asymptomatic bacteriuria; OAB: Overactive bladder; UTI: Urinary tract infection; UUI: Urge urinary incontinence
